# Association Between Caries Risk and Public Water Fluoridation in Balsas, Maranhão: A Cross-Sectional Study

**DOI:** 10.3390/healthcare14111592

**Published:** 2026-06-05

**Authors:** Laura Valentina Borges Pes, Alanna Ramalho Mateus, Haylla de Faria Horta, Adrielle Ouchi Lopes, Brenda Renata Lopes Justo, João Victor de Araújo Narciso, Mariana Gabriel, Sérgio Alves Guida Freitas Júnior, Janaílla Ribeiro Moura, Caio Sampaio, Adolfo José da Mota, Wilson Galhego Garcia, Cristina Antoniali

**Affiliations:** 1 Graduate Program in Sciences, School of Dentistry, São Paulo State University (UNESP), Araçatuba 16015-050, SP, Brazil; laura.pes@unesp.br (L.V.B.P.); alanna.mateus@unesp.br (A.R.M.); haylla.faria@unesp.br (H.d.F.H.); adrielle.ouchi@unesp.br (A.O.L.); j.narciso@unesp.br (J.V.d.A.N.); 2 School of Dentistry, São Paulo State University (UNESP), Araçatuba 16015-050, SP, Brazil; brenda.lopes@unesp.br; 3 Department of Basic Sciences, School of Dentistry, São Paulo State University (UNESP), Araçatuba 16015-050, SP, Brazil; marianagabrielodonto@gmail.com (M.G.); wilson.galhego-garcia@unesp.br (W.G.G.); 4 Municipal Health Department of Balsas, Balsas 65800-000, MA, Brazil; sergio.junior@prof.unibalsas.edu.br; 5 School of Dentistry, UNIBALSAS University Center, Balsas 65800-000, MA, Brazil; janailla.moura@alu.unibalsas.edu.br; 6 Department of Pediatric Dentistry and Public Health, School of Dentistry, São Paulo State University (UNESP), Araçatuba 16015-050, SP, Brazil; caio.sampaio@unesp.br; 7 Graduate Program in Biotechnology, Biosciences Institute, Federal University of Amazonas, Manaus 69080-900, AM, Brazil; adolfo.mot@gmail.com

**Keywords:** dental caries, child, preschool, water fluoridation, drinking water, oral health, public health, social determinants of health

## Abstract

**Highlights:**

**What are the main findings?**

- High prevalence of dental caries was observed among preschool children in Balsas, Maranhão.
- Public water supply showed low fluoride concentrations, characterizing hypofluoridation.

**What are the implications of the main findings?**

- The results highlight the need to strengthen oral health promotion and preventive strategies in early childhood.
- Public policies ensuring adequate water fluoridation are essential to reduce the burden of dental caries.

**Abstract:**

Introduction/Objectives: Dental caries is a significant public health burden in Brazil, with regional disparities deeply affecting children in the North and Northeast. This study assessed the prevalence and severity of dental caries in preschool children from Balsas (MA) and investigated its association with fluoride concentrations in the public water supply. Methods: A cross-sectional study was conducted with 256 children (aged 3–5 years) enrolled in municipal schools. Schools were selected using a stratified sampling strategy based on the water network, with exposure validated via chemical analysis. Calibrated dentists performed examinations using the FOA-UNESP risk scale, assessing biofilm and gingivitis as objective clinical proxies for hygiene. Fluoride levels were analyzed using an ion-selective electrode. Statistical analysis included Fisher’s exact test, Kruskal–Wallis, and Multiple Correspondence Analysis (MCA). Results: Caries prevalence was 60.16%, with 41.8% of children in severe risk categories (F: 27.0%; G: 14.8%). Water analysis revealed universal hypofluoridation (0.02–0.34 µg F/mL). A significant association was found between residual fluoride (0.02 µg F/mL) and greater caries severity (*p* = 0.04). Poor hygiene markers (biofilm and gingivitis) were significantly associated with a higher number of decayed teeth (*p* < 0.05). MCA identified a cluster linking residual fluoridation to severe clinical conditions and social vulnerability. Conclusions: High caries prevalence associated with inadequate fluoridation highlights critical regional health inequalities in Balsas. These findings underscore the urgent need for policies ensuring universal water fluoridation and strengthening school-based preventive strategies to mitigate the impact of social determinants on child development.

## 1. Introduction

Dental caries remains one of the most prevalent chronic diseases worldwide, affecting billions of people and representing a major burden on global public health [1]. In 2021, the World Health Assembly approved a resolution reinforcing the inclusion of oral health in national policies and establishing clear goals for its integration into health systems by 2030 [2]. A particularly critical manifestation is Early Childhood Caries (ECC), defined as the presence of one or more decayed, missing, or filled tooth surfaces in any primary tooth in children younger than six years [3]. ECC is an aggressive condition that can lead to chronic pain, systemic infections, and impaired physical and cognitive development [3].

Community water fluoridation is recognized globally as one of the most effective and equitable public health measures for caries prevention [4,5]. Its mechanism of action is dual: systemically, through ingestion during tooth formation, and topically, by maintaining low, constant levels of fluoride in the oral cavity [4]. This promotes the remineralization of early enamel lesions and inhibits the enzymatic activity of cariogenic bacteria [4]. Global evidence confirms that consistent fluoridation can significantly reduce caries prevalence in children and adolescents [5].

In Brazil, the creation of the National Oral Health Policy (PNSB) in 2004 marked a milestone in reorganizing oral healthcare within the Unified Health System (SUS) [6]. Since then, inequalities in access to dental services have decreased; however, significant regional disparities and vulnerabilities among socially disadvantaged populations persist [7]. Recent epidemiological data are concerning: 46.4% of Brazilian children aged 1 to 9 years present untreated dental caries [8], and the North and Northeast regions show the worst indicators in the country, with a dmft index 63% higher than that of the Southeast [9].

In this context, the literature highlights the need to strengthen primary healthcare through the promotion of self-care, reduction in risk factors, and expanded access to essential oral health services [10]. However, a significant knowledge gap remains regarding oral health in regions of rapid economic expansion, such as the MATOPIBA agricultural frontier. Balsas (MA) is a strategic city in this region, yet epidemiological data on preschool children remains scarce. This study addresses this gap by investigating if public health infrastructure, such as water fluoridation, has kept pace with regional development.

The primary objective of this study was to assess the burden and severity of dental caries in preschool children (aged 3 to 5 years) in Balsas, MA. Secondary objectives included: (I) investigating the association between caries risk and fluoride concentrations in the public water supply; and (II) discussing the implications for regional public health planning and the mitigation of social determinants of health. In addition to evaluating the oral health status of preschoolers, this study aimed to investigate fluoride concentrations in the public water supply, as fluoridation is a fundamental—yet often overlooked—pillar of caries prevention in vulnerable regions.

## 2. Materials and Methods

This was a cross-sectional observational study conducted in Balsas, Maranhão, Brazil. All procedures were performed in accordance with the Declaration of Helsinki and were approved by the Human Research Ethics Committee (CEP), School of Dentistry, Araçatuba, UNESP (Protocol Code: CAAE 66178622.6.0000.5420). Prior to the start of the study, written informed consent was obtained from the parents or legal guardians of all participating children. Furthermore, children provided verbal assent before undergoing clinical examinations.

### 2.1. Contextualization of the Municipality of Balsas (MA)

Balsas is located in southern Maranhão, within the MATOPIBA agricultural frontier (comprising the states of Maranhão, Tocantins, Piauí, and Bahia). According to the 2022 Census, the municipality has 101,616 inhabitants and a per capita GDP of R$ 65,059.77, ranking fifth in the state [11]. The local health infrastructure includes 19 Oral Health Teams linked to the Family Health Strategy (FHS) [12] and the School Health Program (PSE), which provides oral hygiene kits and educational activities.

The study was integrated into the ‘Happy Smile’ Project (Projeto Sorriso Feliz), a university extension initiative developed by the School of Dentistry of UNESP/Araçatuba in partnership with municipal Health and Education Departments. This project focuses on oral health promotion from early childhood through prevention, early diagnosis, and family guidance. In Balsas, the project provided the framework for the study, involving: (i) ludic educational lectures for children and teachers; (ii) supervised toothbrushing sessions; (iii) the distribution of oral hygiene kits (toothbrush and fluoridated toothpaste); and (iv) epidemiological screening for dental caries. Children identified with treatment needs during the screening were formally referred to the municipal Primary Health Care Units (UBS) for clinical intervention.

### 2.2. Implementation of the Happy Smile Project in Balsas

The ‘Happy Smile’ Project was implemented in Balsas through a partnership between the School of Dentistry, Araçatuba, UNESP, the School of Dentistry, UNIBALSAS, and the Municipal Government through its Health and Education Departments. The project targeted children aged 3 to 5 years and 11 months enrolled in the municipal education system. Educational interventions were strictly integrated into the study protocol. Prior to the clinical examinations, standardized meetings were held with parents and legal guardians to provide guidance on reducing free sugar consumption [13] and the mandatory use of fluoridated toothpaste (>1000 ppm F) [14]. These meetings addressed key behavioral determinants of caries risk, ensuring that the study also functioned as a primary prevention platform.

#### Implementation and Intersectoral Coordination

The implementation of the project was based on an intersectoral approach, involving coordinated meetings with municipal stakeholders, including health secretaries, oral health coordinators, social workers, and school coordinators. Crucially, the management of the Autonomous Water and Sewage Service (SAAE) participated in discussions regarding the technical aspects of water fluoridation and the mapping of the distribution network. The coordination of the UNIBALSAS Dentistry Program facilitated the participation of undergraduate students, who assisted in the logistics and educational activities under the direct supervision of the lead dentist (LVBP), ensuring strict adherence to the standardized research protocol.

### 2.3. Team

School visits were conducted between April and June 2023. The clinical team consisted of two dentists (LVBP and SAGFJ), previously trained and calibrated for dental caries diagnosis and risk assessment. The calibration process followed the World Health Organization (WHO) criteria [5] and the FOA-UNESP risk scale. It involved a two-stage process: a theoretical phase for reviewing diagnostic criteria and a practical phase involving the examination of children. High inter-examiner reliability was achieved, with a Kappa index value of 0.79, indicating substantial diagnostic agreement.

Dentistry students from UNIBALSAS participated in educational activities, oral hygiene instruction, and odontogram recording under direct supervision. All students received prior training and participated as volunteers. These measures were implemented to mitigate potential sources of bias by ensuring methodological standardization and high reliability across all individuals involved in data collection.

### 2.4. Patient Selection

According to the Municipal Education Department, 1800 children aged 3 to 5 years and 11 months were enrolled in schools in Balsas, with 1119 distributed across the five evaluated units. The sample size was estimated using Epi Info™ version 7.2 (Centers for Disease Control and Prevention—CDC, Atlanta, GA, USA); however, all eligible children were invited to participate, resulting in a final sample of 256 children.

The selection of schools followed a stratified sampling strategy based on the municipal water distribution network to represent different exposure profiles: units connected to the central Water Treatment Station (ETA) at varying distances and those supplied by independent artesian wells. Crucially, this geographic selection was formally validated through direct chemical analysis of fluoride concentrations in water samples collected at each school (as detailed in Section 2.6), ensuring an accurate and objective classification of exposure levels.

The selection of children from municipal public schools ensures a relatively homogeneous socioeconomic profile, characteristic of the low-to-middle income population in the region, which helps minimize socioeconomic status as a confounding variable in the analysis.

Prior meetings were held with parents or legal guardians to provide guidance on oral hygiene, use of fluoridated toothpaste, and sugar consumption, as well as to explain and obtain signed Free and Informed Consent Forms (FICF).

Clinical evaluations were conducted between April and June 2023 by the dentists (LVBP and SAGFJ). Inclusion criteria were healthy children aged 3 to 5 years and 11 months enrolled in schools with signed consent forms. The informed consent form was provided to the parents or legal guardians of all participating children for their information, and written informed consent was obtained from the parents or legal guardians prior to participation in the study. Exclusion criteria included absence of consent or failure to attend the examination. For children who resisted examinations, parents and teachers assisted during the procedure. The number of participants per school is presented in Table 1.

### 2.5. Caries Risk Classification

**Figure 1:**
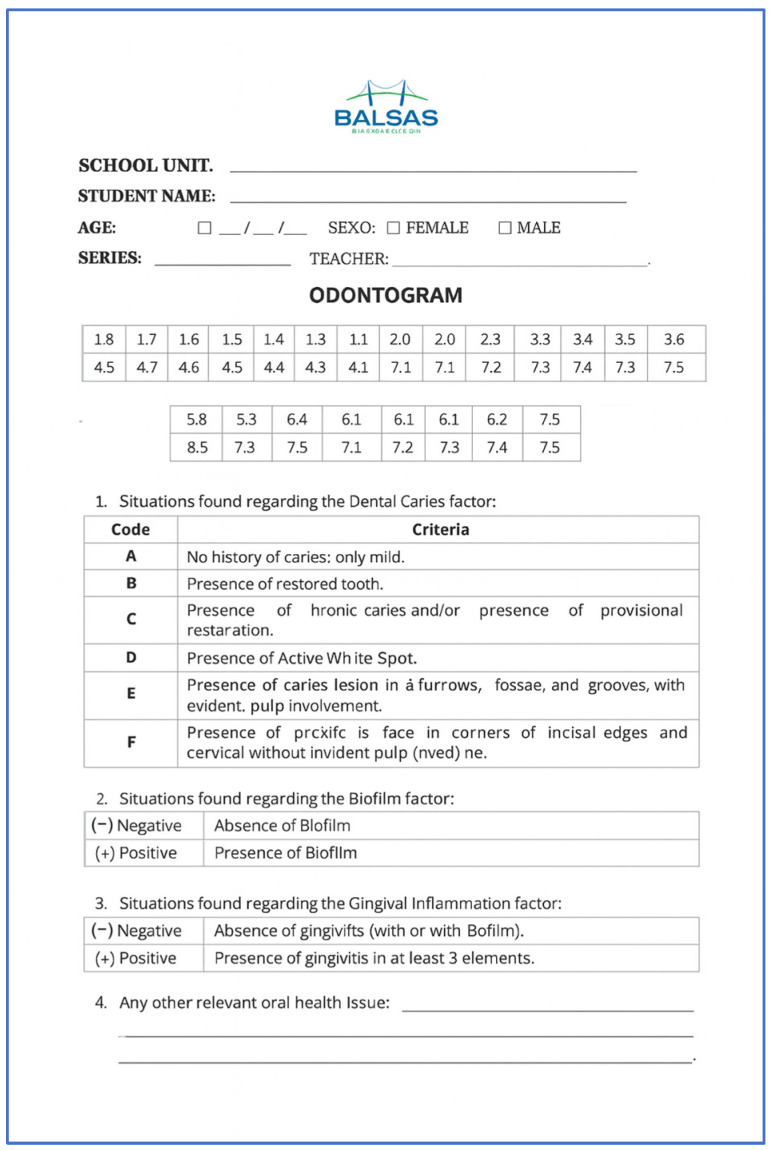
Odontogram used in the clinical evaluation (caries diagnosis) of children.

Clinical examinations were performed in school settings under natural lighting and ventilation, using disposable wooden spatulas and plane mirrors. The diagnostic criteria followed the visual-tactile method for dental caries detection. Risk assessment was documented using a standardized odontogram (Figure 1) developed by the faculty team of FOA-UNESP, based on the risk classification model proposed by the Guidelines of the State Policy for Oral Health Care of the São Paulo State Health Department.

For each tooth examined, the clinical condition was classified from A to G as follows: Category A represents sound teeth; Category B, restored teeth; Category C, chronic lesions or provisional restorations; Category D, active white spot lesions; and Categories E and F, cavitated lesions in pits, fissures, or smooth surfaces without pulpal involvement. Category G was defined as the “most severe clinical condition”, characterized by extensive cavitated lesions with clinical signs of pulpal or periapical involvement (e.g., pulpitis, abscess, or exposed pulp). This detailed scale ensures diagnostic reproducibility and aligns local metrics with international clinical standards.

Oral hygiene and biological risk were objectively assessed using the ‘Biofilm Factor’ and ‘Gingivitis Factor’ as clinical proxies. The presence of visible biofilm was utilized as a marker for recent hygiene efficiency, while gingival inflammation served as a biological indicator of prolonged plaque accumulation. This clinical approach was intentionally preferred over self-reported questionnaires to mitigate recall and social desirability biases.

During the examinations, findings were dictated to a trained undergraduate student who assisted in the recording process (Supplementary Material, Figure S1). All personnel used appropriate personal protective equipment. Following the clinical evaluation, children classified between C and G were referred for dental treatment at the Primary Health Care Units closest to their residence. Referrals were prescribed by the examining dentist and delivered to the parents or guardians through the school teachers.

### 2.6. Analysis of Fluoride Concentration in Water

Water samples were collected in duplicate from the five participating schools and the municipal Water Treatment Station (ETA) in Balsas, synchronized with the period of clinical examinations. Each 15 mL sample was stored in new polypropylene tubes, previously labeled and kept in a dry, light-protected environment until analysis. The quantification of fluoride concentration [F^−^] was conducted at the Pediatric Dentistry Research Laboratory of the School of Dentistry (FOA-UNESP).

For the analytical procedure, 1.0 mL of each sample was buffered with 1.0 mL of TISAB II (Total Ionic Strength Adjustment Buffer; Orion), ensuring a 1:1 ratio to provide constant ionic strength and release complexed fluoride ions. Readings were performed using a fluoride ion-selective electrode (Orion 9409) and a reference electrode (Orion 900100) coupled to a digital potentiometer (Orion, Thermo Fisher Scientific, Waltham, MA, USA). The equipment was calibrated using a series of standard solutions ranging from 0.25 to 4.0 µg F/mL, prepared by serial dilution of a 100 ppm F stock solution (Orion 940907). Millivolt values (mV) were recorded and converted to µg F/mL using Microsoft Excel™ (version 2010), maintaining a linear calibration curve (r^2^ > 0.99). All samples and standards were analyzed in duplicate to ensure analytical precision and reproducibility.

### 2.7. Data Analysis

Statistical analyses were specifically designed to correlate clinical caries data with water fluoridation levels, identifying whether public supply deficiencies were associated with the severity of the disease in the sample.

Quantitative variables were summarized using measures of central tendency and dispersion, presented via boxplots. Pearson’s correlation coefficient assessed the relationship between age and the number of decayed teeth, following standard interpretation criteria for strength of association. Qualitative variables were described using proportions and bar graphs.

To investigate the multifactorial nature of dental caries, inferential statistical methods were employed. Associations between qualitative variables, including clinical proxies (biofilm and gingivitis) and caries risk, were examined using Multiple Correspondence Analysis (MCA). Age and the number of decayed teeth were categorized according to Sturges’ rule for this analysis. Due to violations of normality assumptions, non-parametric tests were utilized. The Mann–Whitney test compared two independent groups, while the Kruskal–Wallis test, followed by Dunn’s post hoc test, was applied for multiple comparisons (*p* < 0.05). Finally, the association between specific water fluoridation levels (hypofluoridated vs. residual) and caries risk severity was evaluated using Fisher’s exact test.

## 3. Results

The epidemiological survey revealed a high burden of disease among preschoolers in Balsas, with an overall dental caries prevalence of 60.2% (*n* = 154).

**Figure 2:**
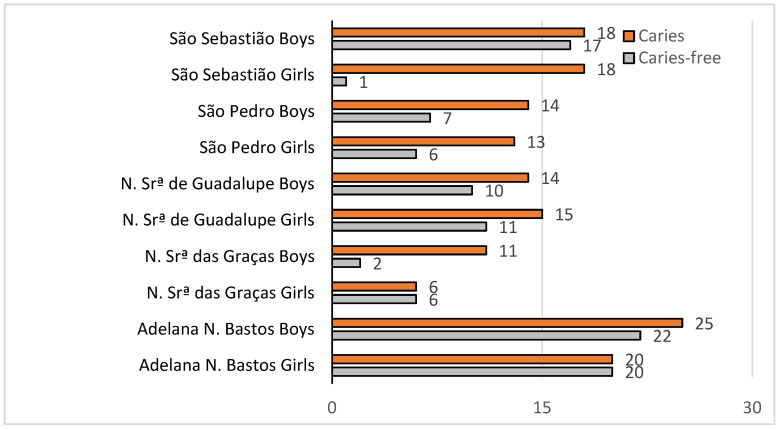
Absolute distribution of preschool children (*n* = 256) with and without dental caries across five municipal schools in Balsas, Maranhão, Brazil, stratified by gender. The term ‘São Sebastião Boys’ indicates the number of male students at São Sebastião School. The same labeling convention is applied to other schools and to female students throughout the figure.

Risk factors appeared equally distributed across genders, as no significant disparities were observed between boys (58.6%) and girls (62.1%), indicating that environmental and behavioral determinants affect the population homogeneously. Analysis by age group identified a clear cumulative risk effect, with the frequency of lesions peaking in the 60–71 months group (50% of cases). This upward trend suggests a failure of existing preventive measures to stabilize the disease before the transition to mixed dentition. Furthermore, as illustrated in Figure 2, the high prevalence across almost all school units confirms that dental caries represents a systemic public health challenge within the municipality rather than a localized issue.

**Figure 3:**
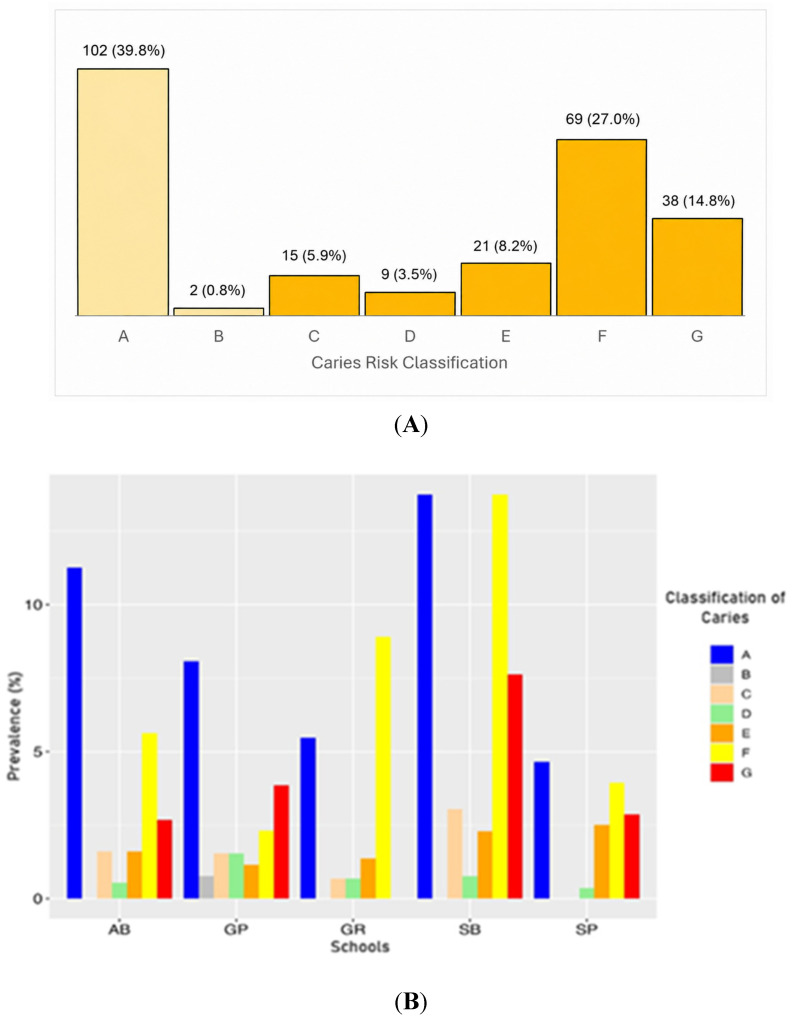
(**A**). Distribution of preschool children (*n* = 256) according to the caries severity and risk classification scale (A–G) in Balsas, Maranhão, Brazil. Category A denotes sound dentition, while Categories F and G represent advanced cavitation and pulpal or periapical involvement, respectively. (**B**). Prevalence of caries risk categories (A–G) stratified by municipal school unit in Balsas, Maranhão, Brazil. School names abbreviations: AB (Adelana N. Bastos); GP (Nossa Senhora de Guadalupe); GR (Nossa Senhora das Graças); SB (São Sebastião); SP (São Pedro).

The assessment of disease severity through the A–G classification revealed a concerning clinical profile among the preschoolers (Figure 3A). Although 39.8% (*n* = 102) of the children were classified as sound (Category A), a significant majority of those affected by the disease presented advanced stages of destruction. Specifically, 41.8% of the total sample fell into the most severe categories: 27.0% in Category F (cavitated lesions on smooth or proximal surfaces) and 14.8% in Category G (pulpal or periapical involvement). This high concentration of severe cases indicates a critical lack of early diagnosis, as nearly 70% of the children with caries already exhibited extensive cavitation or signs of pulpal involvement.

The spatial distribution of these findings (Figure 3B) highlights regional disparities within the municipality. While schools like Adelana Noleto Bastos (AB) and Nossa Senhora de Guadalupe (GP) showed the highest proportions of sound dentition, the São Sebastião (SB) unit presented a critical cluster of severity, with the highest combined frequency of F and G classifications. This variation suggests that localized environmental determinants or differences in access to primary dental care across school territories may be influencing the clinical progression of the disease.

The analysis of the disease burden revealed a high intensity of dental caries among the affected preschoolers. Most children exhibited multiple lesions, with the 5–9 decayed teeth range being the most frequent. Interestingly, the Multiple Correspondence Analysis (MCA) and the low Pearson correlation coefficient (ρ = 0.14) indicated that the number of decayed teeth and caries risk were not strictly associated with age in this sample. This suggests that other biological and environmental factors, rather than merely the duration of exposure (age), are the primary drivers of disease progression in this population.

**Figure 4:**
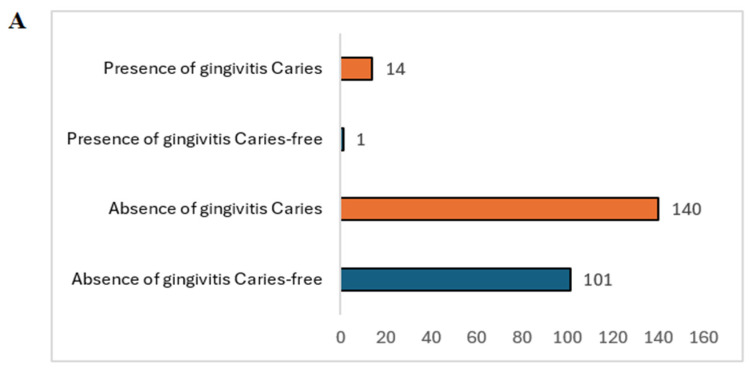

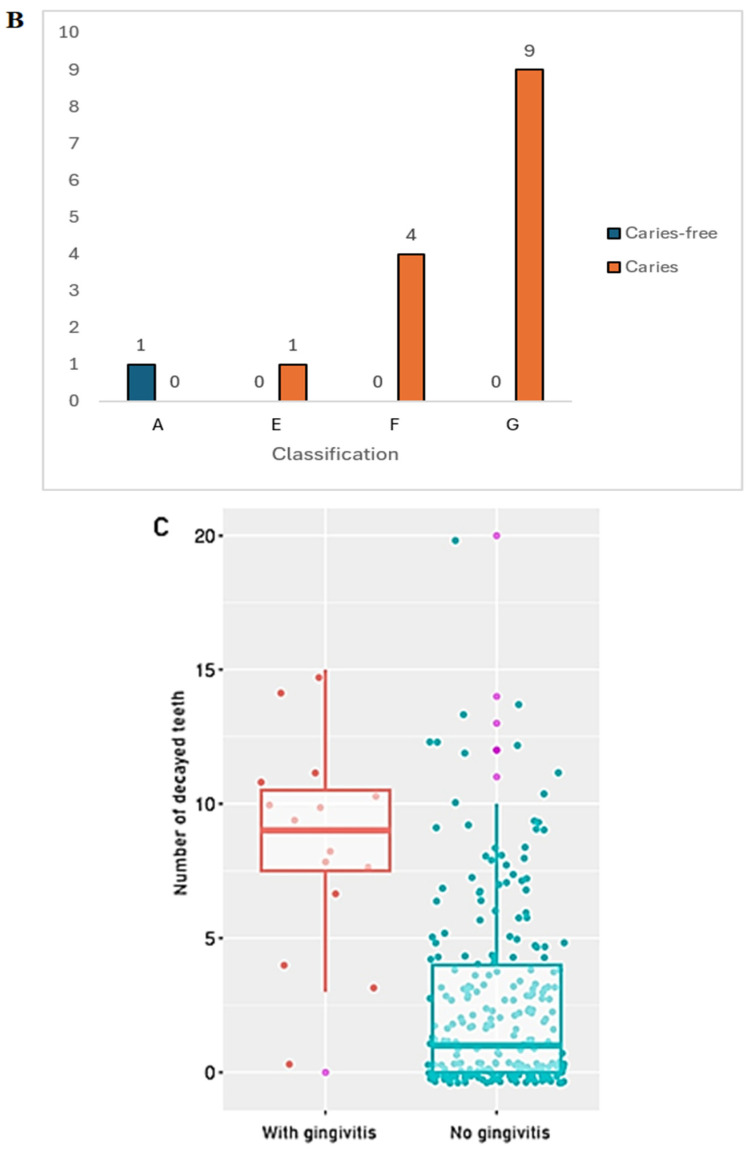
Association between gingivitis, dental caries prevalence, and disease burden among preschoolers (*n* = 256) in Balsas, MA, Brazil. (**A**) Absolute distribution of children with and without dental caries stratified by the presence or absence of gingivitis. (**B**) Distribution of caries severity levels (Scale A–G) specifically among children diagnosed with gingivitis. (**C**) Boxplot comparing the number of decayed teeth between children with and without gingivitis, highlighting a statistically significant difference (*p* < 0.05).

Gingivitis, used as a clinical proxy for prolonged biofilm accumulation, showed a low overall prevalence (~6.0%). However, its presence was a strong indicator of high disease severity. As illustrated in Figure 4A,B, almost all children with gingivitis also presented with dental caries, predominantly classified in the most severe category (Category G). Most importantly, the inferential analysis (Figure 4C) confirmed that children with gingivitis had a significantly higher number of decayed teeth compared to those without the condition (*p* < 0.05). This finding underscores the role of oral hygiene as a critical biological determinant: although gingivitis is less frequent than caries, its occurrence identifies a high-risk subgroup with a disproportionately higher burden of untreated decay.

**Figure 5:**
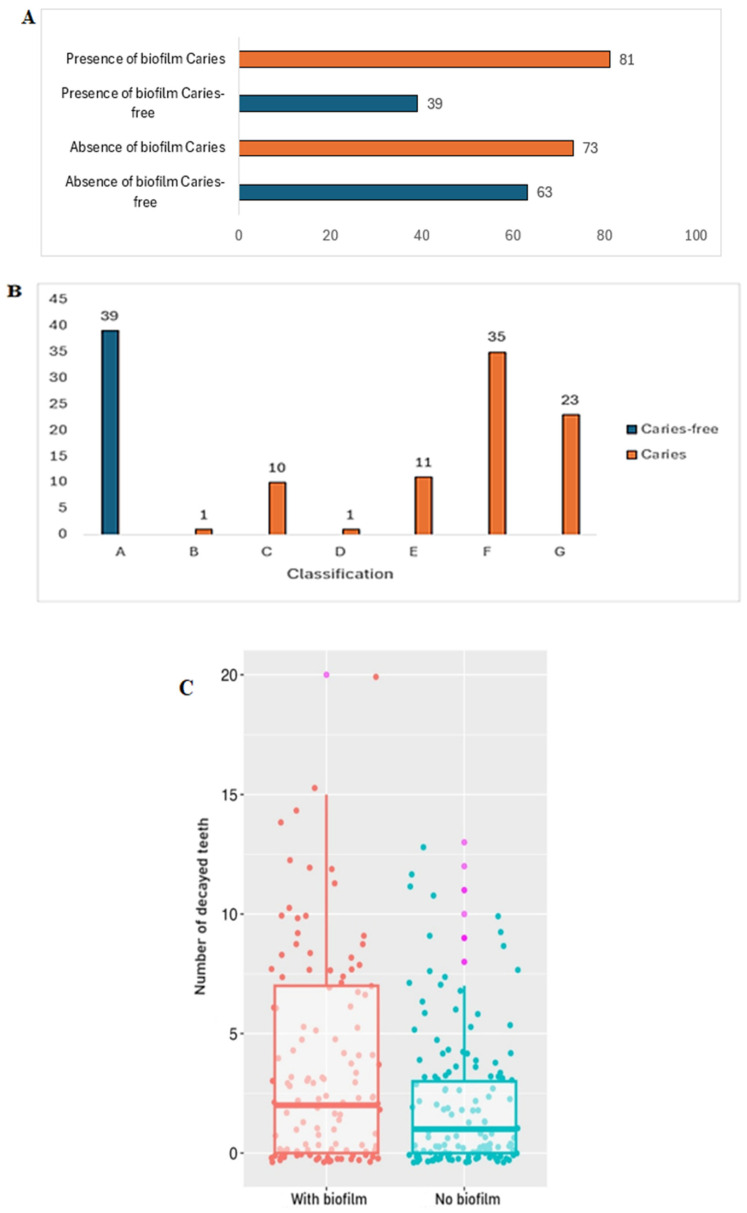
Influence of dental biofilm on caries prevalence and severity among preschoolers (*n* = 256) in Balsas, MA, Brazil. (**A**) Distribution of children with and without dental caries stratified by the presence of visible biofilm. (**B**) Caries severity levels (Scale A–G) specifically among children with biofilm. (**C**) Comparative analysis of the number of decayed teeth between children with and without biofilm, showing a statistically significant difference (*p* < 0.05).

The presence of dental biofilm, utilized as a clinical marker for recent oral hygiene efficiency, was identified in 46.9% of the children (*n* = 120), with identical distribution between genders. An analytical comparison revealed that children with visible biofilm had a significantly higher prevalence of dental caries compared to those with clean tooth surfaces (Figure 5A). Beyond prevalence, the presence of biofilm was a strong indicator of disease progression; as illustrated in Figure 5B, children with poor hygiene were predominantly classified into the most advanced stages of severity (Categories F and G). Furthermore, the inferential analysis confirmed that the mean number of decayed teeth was significantly higher in the group with biofilm (*p* < 0.05), reinforcing that inadequate biofilm control is a primary driver of the high disease burden observed in this population.

**Figure 6:**
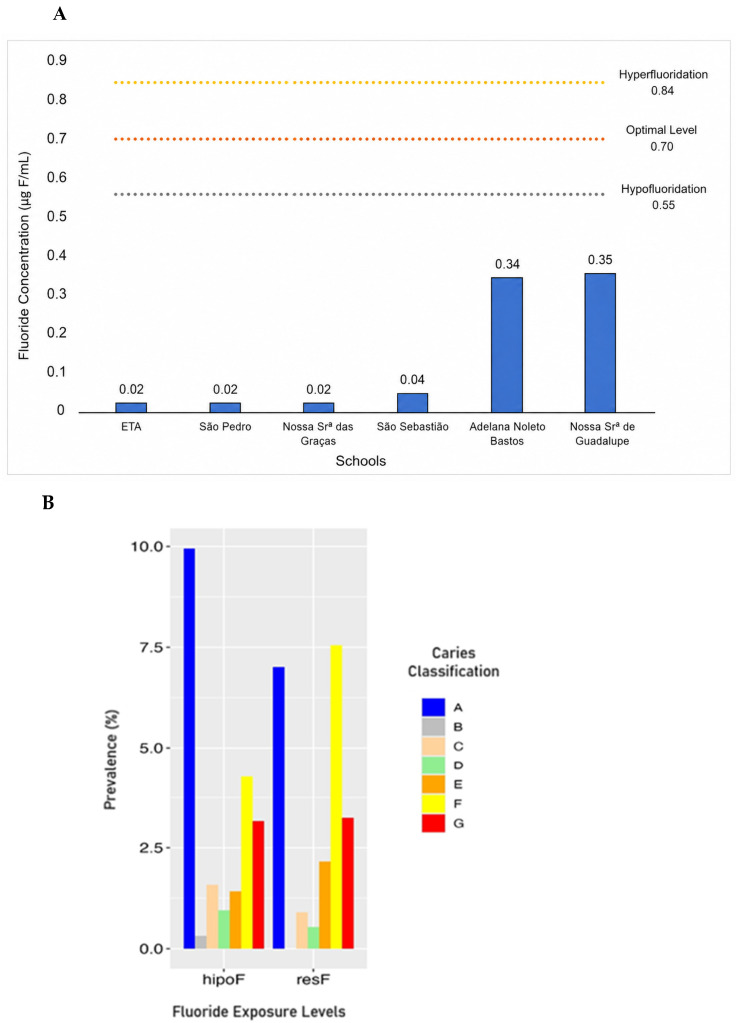

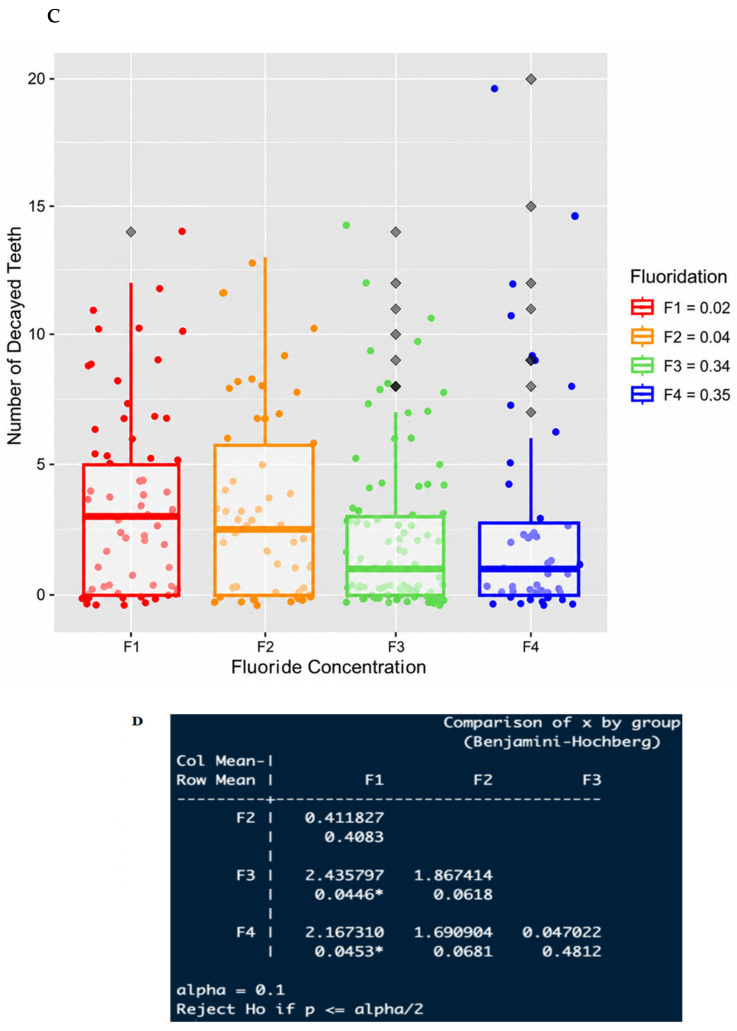
Analysis of public water fluoridation and its association with dental caries among preschoolers (*n* = 256) in Balsas, MA, Brazil. (**A**) Fluoride concentration (µg F/mL) at the Water Treatment Station (ETA) and the schools compared to international quality thresholds. (**B**) Prevalence of caries severity (Scale A–G) stratified by fluoride exposure levels (Hypofluoridated vs. Residual). (**C**) Boxplot comparing the number of decayed teeth across four fluoride concentration levels (F1: 0.02; F2: 0.04; F3: 0.34; F4: 0.35 µg F/mL). (**D**) Pairwise comparison matrix (Dunn’s post-hoc test with Benjamini-Hochberg adjustment) for the number of decayed teeth; asterisks (*) indicate statistically significant differences at *p* < 0.05.

Chemical analysis revealed that public water fluoridation in Balsas is critically deficient. All samples collected from the Water Treatment Station (ETA) and school units were below the minimum recommended threshold (0.55 µg F/mL), characterizing a scenario of universal hypofluoridation (Figure 6A). Notably, the ETA and three school units (São Pedro, Nossa Senhora das Graças, and São Sebastião) exhibited only residual fluoride levels (0.02 to 0.04 µg F/mL). In contrast, the Adelana Noleto Bastos and Nossa Senhora de Guadalupe units showed relatively higher concentrations (0.34 to 0.35 µg F/mL), although still categorized as hypofluoridated.

This variation in fluoride exposure, despite being entirely below the optimal range, was significantly associated with the clinical outcomes. As shown in Figure 6B, children in schools with low hypofluoridation (0.34–0.35 µg F/mL) presented a better preventive profile, with a higher prevalence of sound dentition (Categories A and B) compared to those exposed only to residual fluoride (*p* = 0.04, Fisher’s exact test). Conversely, advanced decay (Category F) was more frequent in schools with residual levels, showing a trend toward clinical significance (*p* = 0.07).

The impact of fluoride concentration on the disease burden was further confirmed by the inferential analysis of the number of decayed teeth (Figure 6C). The Kruskal–Wallis test identified a significant difference in the distribution of decay across the exposure groups (*p* < 0.05). Specifically, the post hoc matrix (Figure 6D) revealed that children exposed to 0.34 µg F/mL (F3) and 0.35 µg F/mL (F4) had a significantly lower number of decayed teeth compared to those in the residual fluoride group (F1 = 0.02 µg F/mL), with *p*-values of 0.044 and 0.045, respectively. These results underscore that even sub-optimal concentrations of fluoride in the public water supply may exert a protective effect against caries progression in high-risk populations.

**Figure 7:**
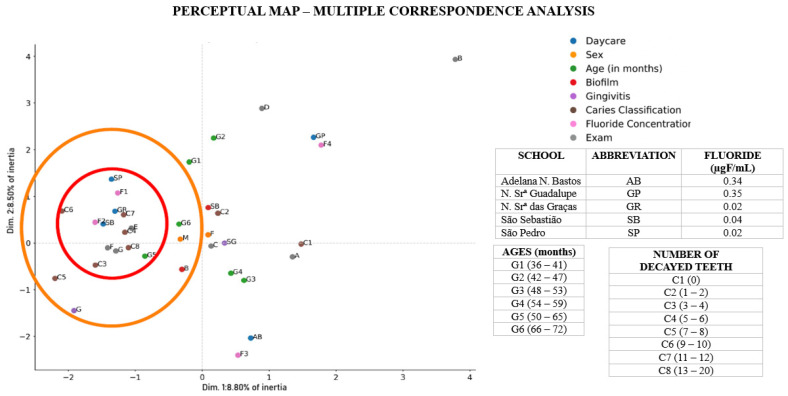
Perceptual map from the Multiple Correspondence Analysis (MCA) illustrating the multivariate associations between fluoride exposure, clinical indicators, and demographic variables among preschoolers (*n* = 256). The red circle highlights the core high-risk cluster, showing the association between residual fluoride levels (F1, F2) and high caries severity/burden. The orange circle represents a broader risk profile, incorporating biological (biofilm, gingivitis) and demographic (age, sex) determinants. Abbreviations: Caries classification (A–G); Number of decayed teeth (C1–C8); Age groups (G1–G6); Fluoride levels (F1–F4); Schools (AB, GP, GR, SB, SP).

The multivariate interplay between environmental, biological, and demographic factors was explored using Multiple Correspondence Analysis (Figure 7). The perceptual map revealed a distinct high-risk cluster (red highlight), identifying a strong synergistic relationship between residual fluoridation levels (F1 and F2: 0.02–0.04 µg F/mL) and the most critical clinical outcomes. This cluster concentrated the highest burden of disease (Categories C3 to C8, representing up to 20 decayed teeth) and the most severe caries classifications (E, F, and G), primarily involving children from the São Pedro, Nossa Senhora das Graças, and São Sebastião schools.

In a broader dimension (orange highlight), this vulnerability profile was further associated with poor oral hygiene indicators, such as the presence of biofilm and gingivitis, as well as the male sex and older age groups (G5 and G6, 50–72 months). Conversely, a protective profile was observed on the opposite side of the perceptual map, where higher (though still sub-optimal) fluoride levels (F3 and F4, 0.34–0.35 µg F/mL) gravitated toward sound dentition (Category A) and a lower number of decayed teeth (C1). These findings indicate that the severity of dental caries in Balsas is not determined by a single factor, but by a multidimensional confluence of insufficient water fluoridation and inadequate biofilm control, which together exacerbate the disease progression as children age.

## 4. Discussion

The main conclusion of this study is the critical deficiency in public water fluoridation in Balsas and its direct association with the high severity of caries in preschoolers. Although community water fluoridation (CWF) is globally recognized as the most cost-effective public health measure for caries prevention [3,15], our analysis revealed a scenario of universal hypofluoridation. Even in this deficient context, the distinction between “residual” (0.02 µg F/mL) and “low hypofluoridation” levels (0.34 to 0.35 µg F/mL) proved clinically significant.

Children exposed to slightly higher concentrations showed a better preventive profile and a lower disease burden (*p* < 0.05), reinforcing the premise that even suboptimal fluoride levels can exert a protective effect [16]. However, the widespread prevalence of advanced lesions (Categories F and G) highlights the failure of current municipal policies to maintain optimal fluoride levels. The situation in Balsas reflects international cases, such as in Windsor, Canada, where the discontinuation or inadequacy of fluoridation led to a rapid increase in childhood caries [17]. These findings highlight a failure in the governance of basic sanitation services, making it necessary to implement “heterocontrol”—independent technical monitoring of fluoride levels—to ensure the reliability and transparency of this collective health measure [18]. In this study, oral hygiene and biological risks were assessed using clinical indicators, biofilm, and gingivitis, rather than self-report questionnaires, which are often subject to recall and social desirability biases. The presence of biofilm, an objective indicator of the effectiveness of oral hygiene, showed a strong association with caries, particularly among children classified in the higher-risk categories F and G (*p* < 0.05). A similar pattern was observed for gingivitis, indicating that gingival inflammation and biofilm accumulation serve as critical biological markers of lesion severity in this population.

These findings reinforce that, although water fluoridation is a collective pillar for prevention [19], individual clinical factors remain decisive in the development of the disease. Furthermore, although the absence of individual food diaries is a limitation, the educational interventions carried out with parents during the ‘Happy Smile Project’ specifically addressed reducing sugar consumption and using fluoride toothpaste. The use of objective clinical markers, such as biofilm, partially compensates for this limitation, providing a tangible record of the interaction between diet and hygiene habits over time [18,20,21,22,23,24,25]. The epidemiological profile identified in Balsas is inseparable from its social determinants. In addition, preventive oral health initiatives based on educational and university–community approaches have demonstrated relevance in promoting oral health and preventive care in childhood [26,27]. The Fisher-Owens conceptual model highlights that caries is influenced not only by classic microbiological and dietary factors, but also by social determinants of health (SDH) at the child, family, and community levels [28].

Our findings, which demonstrate greater severity in schools with lower fluoride levels and specific socioeconomic challenges, reinforce the critical role of Social Determinants of Health (SDH) in the epidemiological profile of Balsas, a municipality in Maranhão—the state with the lowest Human Development Index (HDI) in Brazil [20,21,29]. From a global perspective, these findings contribute to the ongoing debate on health equity and governance in developing regions. Challenges related to fluoridation surveillance and public health governance have been widely discussed [19,30]. In a world where the WHO Global Oral Health Report of 2022 advocates a shift towards population-based preventive measures [31], the case of Balsas serves as a warning: the expansion of water treatment infrastructure is insufficient if it is not accompanied by rigorous quality surveillance and a political commitment to community-based prevention.

Although these results are significant, this study has limitations. Because it captures only a specific moment in time (cross-sectional), we cannot definitively establish cause and effect. Also, since we focused on a single city, the findings may not apply to all regions. Future research should follow children over longer periods to see how water fluoridation, better hygiene, and parental guidance work together. Long-term studies are essential to confirm if these combined prevention programs truly work in vulnerable communities.

## 5. Conclusions

Dental caries prevalence in Balsas is high and directly linked to public water hypo-fluoridation. It is imperative to implement public policies that ensure optimal fluoride levels through independent technical monitoring. Furthermore, integrating academic initiatives like “Projeto Sorriso Feliz” with municipal health services is essential to expand access to preventive care, increase the visibility of children’s oral health, and promote health equity in the region.

## Figures and Tables

**Table 1:** Number of enrolled and evaluated children in the Municipal Early Childhood Education Schools of Balsas, MA.

Schools	Enrolled	Evaluated
**Adelana Noleto Bastos**	373	87
**Nossa Senhora Aparecida**	146	25
**Nossa Senhora de Guadalupe**	260	50
**São Pedro**	279	40
**São Sebastião**	131	54

## Data Availability

The data used in this study contains sensitive information about children and are protected by ethical requirements. Therefore, they cannot be made publicly available. Anonymized datasets may be provided upon reasonable request to the corresponding author, subject to approval by the Research Ethics Committee responsible.

## Supplementary Materials

The following supporting information can be downloaded at: https://www.mdpi.com/article/10.3390/healthcare14111592/s1. Figure S1. Caries Risk and Management Classification.

## Abbreviations

The following abbreviations are used in this manuscript:

AB	Adelana Noleto Bastos School
GP	Nossa Senhora de Guadalupe School
GR	Nossa Senhora das Graças School
SB	São Sebastião School
FHS	Family Health Strategy
ETA	Water Treatment Station (ETA, from the Portuguese *Estação de Tratamento de Água*).
PSE	School Health Program (PSE, from the Portuguese *Programa Saúde na Escola*)
UBS	Primary Health Care Units (UBS, from the Portuguese *Unidades Básicas de Saúde*)
SAAE	Autonomous Water and Sewage Service (SAAE, from the Portuguese *Serviço Autônomo de Água e Esgoto*)
UNESP	São Paulo State University
UNIBALSAS	University Center of Balsas
CEP	Human Research Ethics Committee (CEP, from the Portuguese *Comitê de Ética em Pesquisa*)
CAAE	Certificate of Presentation for Ethical Consideration (CAAE, from the Portuguese *Certificado de Apresentação para Apreciação Ética*)
MCA	Multiple Correspondence Analysis
