# Supplementary material for: Association Between Caries Risk and Public Water Fluoridation in Balsas, Maranhão: A Cross-Sectional Study
Source: Healthcare (Basel). 2026 Jun 5;14(11):1592. doi: 10.3390/healthcare14111592 (PMC13257183; doi:10.3390/healthcare14111592)
Supplement: Supplementary file 1 [file healthcare-14-01592-s001.zip › healthcare-4239579-supplementary.pdf]

CODE	CARIES FACTOR	BIOFILM FACTOR	GENGIVITIS FATOR	RISK LEVEL
A -	**A –** No history of caries; all teeth sound.	-	-	Low
A +		+	+	High
B -	**B –** Presence of restoration.	-	-	Moderate
B +		+	+	High
C -	**C –** Presence of chronic caries lesion and/or temporary restoration.	-	-	Moderate
C +		+	+	High
D -	**D –** Active white spot lesion.	-	-	High
D +		+	+	
E -	**E –** Pit and fissure caries without evident pulpal involvement.	-	-	High
E +		+	+	
F -	**F –** Proximal lesion, incisal angle lesion, or cervical third lesion without evident pulpal involvement.	-	-	
F +		+	+	
G -	**G –** Suspected pulpal or periapical involvement (pulpitis, fistula, exposed pulp, abscess, residual focus, pain).	-	-	
G +		+	+	

Figure S1. Caries Risk and Management Classification.
